# Wearable and Invisible ECG Quality and Usability Assessment in Cockpit Monitoring

**DOI:** 10.3390/bios16090486

**Published:** 2026-09-02

**Authors:** Mariangela Pinnelli, Ana Sofia Antunes Calado, Tiago Filipe Rodrigues Fernandes, Paulo Sérgio de Brito André, Emiliano Schena, Carlo Massaroni, Hugo Plácido da Silva

**Affiliations:** 1 Department of Engineering, Università Campus Bio-Medico di Roma, 00128 Rome, Italy; mariangela.pinnelli@unicampus.it (M.P.); e.schena@unicampus.it (E.S.); 2 Instituto de Telecomunicações, Instituto Superior Técnico, 1049-001 Lisbon, Portugal; ana.calado@tecnico.ulisboa.pt (A.S.A.C.); tiago.filipe.rodrigues.fernandes@tecnico.ulisboa.pt (T.F.R.F.); paulo.andre@tecnico.ulisboa.pt (P.S.d.B.A.); hugo.placido.silva@tecnico.ulisboa.pt (H.P.d.S.); 3 Fondazione Policlinico Universitario Campus Bio-Medico di Roma, 00128 Rome, Italy

**Keywords:** cockpit monitoring, electrocardiography, invisible ECG, printed electrodes, heart rate estimation, signal quality assessment, wearable ECG, pilot workload

## Abstract

Continuous physiological monitoring can support pilot-state assessment, but routine cockpit use requires sensing approaches that are both unobtrusive and physiologically reliable. Wearable ECG (wECG) provides stable cardiac recordings through skin-contact electrodes, whereas cockpit-integrated invisible ECG (iECG) can reduce user burden by acquiring signals through instrumented controls. However, iECG depends on intermittent hand contact and may show incomplete ECG morphology even when cardiac timing information is still preserved. This study proposes a window-based framework to assess the quality and task-specific usability of simultaneous wECG and iECG acquired during simulated flight. ECG data were collected in an Airbus A320 simulator from 14 volunteers, including experienced pilots and novices. The framework combines contact availability, R-peak reliability, PQRST morphology, and complementary signal quality indices into graded usability classes for heart rate (HR)-oriented monitoring. The iECG channel remained accessible for most of the analyzed recording time, with 93.8% of windows showing full or partial contact. Several windows classified as low quality by individual SQIs were retained as HR-usable when contact and R-peak timing remained reliable, indicating that single-metric rejection can be overly conservative for external-contact ECG. HR agreement supported the physiological relevance of the proposed classes: concordant wECG–iECG windows showed a mean absolute error (MAE) of 1.1 bpm, compared with 7.1 bpm for discordant windows. Bland–Altman analysis for iECG windows classified as usable for HR estimation showed a small mean bias of 1.4 bpm. These findings indicate that incomplete ECG morphology does not necessarily imply loss of HR usability, and that contact-aware, task-specific classification can preserve useful physiological information from unobtrusive cockpit interfaces.

## 1. Introduction

Real-time physiological monitoring is increasingly relevant in safety-critical environments, where physiological signals can support objective assessment of operator state during demanding tasks [1,2,3,4]. In civil aviation, ECG is particularly relevant because it provides cardiac timing information while remaining compatible with ongoing task execution. While commercial aviation remains one of the safest transportation sectors, recent International Air Transport Association (IATA) and International Civil Aviation Organization (ICAO) safety reports show that accident rates still fluctuate across years, despite increasing traffic volumes and very low overall risk [5,6,7]. Beyond accident statistics alone, these reports reinforce the continued relevance of human factors, including fatigue, workload, stress, decision making, and task execution, especially during demanding flight phases. Within this perspective, physiological monitoring is not intended to replace existing safety barriers, but to provide an additional objective layer for pilot-state assessment during real operational demands [8].

Among physiological signals, electrocardiography (ECG) is particularly relevant for pilot-state monitoring because it enables the extraction of heart rate (HR), R-to-R (RR) intervals, and, when signal quality is sufficient, heart rate variability (HRV). These parameters provide indirect but meaningful information on autonomic regulation and psychophysiological activation, and have been widely used to investigate workload, stress, vigilance, fatigue, and recovery in aviation and other high-demand scenarios [9,10,11]. In this context, the aim of ECG monitoring is not necessarily full diagnostic interpretation of the waveform, but the reliable extraction of cardiac timing information. This distinction is important because HR and RR-derived metrics may remain usable even when complete PQRST morphology is partially degraded.

Traditionally, ECG acquisition relies on skin-contact electrodes, often based on pre-gelled Ag/AgCl interfaces, which provide stable and low-impedance recordings and are therefore well suited for clinical monitoring and reference measurements. However, this configuration is less compatible with routine operational use, where skin preparation, adhesive electrodes, lead wires, and body-mounted devices may increase setup time, reduce comfort, and interfere with natural activity. For this reason, recent research has moved toward less obtrusive ECG solutions, including dry electrodes, textile or printed interfaces [12,13], and other wearable (wECG) configurations [14,15,16]. Printed sensing approaches are particularly relevant because they can be implemented as skin-contact sensing elements integrated into garments [17,18], or objects [19,20], supporting more natural interaction with the monitoring system. These technologies aim to preserve physiologically useful information while reducing the burden imposed on the operator.

A further step toward unobtrusive monitoring is represented by invisible ECG (iECG) systems, where electrodes are integrated into objects or surfaces naturally contacted by the user. Capacitive and external-contact approaches have been investigated in chairs, beds, backrests, vehicle seats, and other human-interface surfaces [21,22,23,24,25]. This concept is particularly relevant for ECG monitoring in operational environments, where physiological monitoring should be compatible with natural activity and should not require the operator to wear additional devices or modify task execution.

However, unlike conventional wECG, the contact between the body and the sensing interface is not guaranteed over time. In embedded or object-integrated iECG, contact may be continuous, partial, or temporarily absent depending on posture, pressure, movement, and interaction with the instrumented surface [8]. Therefore, before assessing ECG signal quality, it is necessary to determine whether the acquired window actually contains physiological information and should not be automatically discarded.

The physiological structure of the ECG waveform explains why signal quality should be interpreted according to the intended analytical task. In standard ECG interpretation, morphology-dependent analysis requires the reliable identification of the P wave, QRS complex, and T wave, together with physiologically plausible temporal relationships such as PR, QRS, and QT intervals [26,27]. This level of morphological completeness is essential when the objective is clinical waveform interpretation. However, HR estimation relies mainly on the temporal distance between consecutive R-peaks, which are associated with ventricular depolarization and are usually the most prominent and sharply defined components of the ECG waveform [28]. Therefore, an ECG segment with degraded PQRST morphology may still retain useful cardiac timing information, provided that R-peaks are consistently detectable and the resulting RR intervals remain physiologically plausible.

This distinction becomes critical when ECG is acquired through dry, textile, capacitive, or external-contact interfaces, where morphology may be distorted by unstable coupling, motion artifacts, variable contact pressure, or changes in electrode position. In these conditions, applying a strictly morphology-oriented criterion may lead to the rejection of windows that are unsuitable for full ECG interpretation but still usable for HR estimation. Conversely, windows affected by missing contact, unreliable beat detection, or implausible RR dynamics should be rejected or assigned to low-confidence classes, even when residual waveform components are visible. Thus, the problem is not only to classify ECG quality in a conventional sense, but to determine whether each segment preserves enough physiological information for the target output.

Several ECG signal quality indices (SQIs) have been proposed to quantify the reliability of ECG recordings in noisy, wearable, and ambulatory conditions. These indices assess complementary properties such as spectral content, statistical distribution, beat-to-beat consistency, detector agreement, and morphology preservation [29,30,31,32]. Morphology-oriented indices, including the dynamic signal quality index (dSQI), are particularly useful for evaluating the similarity and consistency of beat-aligned ECG waveforms [33]. They are therefore well suited to identify windows with stable PQRST morphology and have been successfully applied to single-lead wearable ECG recordings acquired through relatively stable interfaces [34]. However, in cockpit-integrated iECG, morphology degradation may occur without complete loss of R-peak timing information, making morphology-based quality alone insufficient to decide whether a window is usable for HR monitoring.

Other approaches, such as signal-to-noise ratio (SNR) estimation or denoising-based evaluation, provide additional information on the spectral cleanliness or recoverability of corrupted ECG signals [35,36,37]. Nevertheless, these measures do not fully solve the task-specific usability problem. A segment may show reduced SNR while still allowing robust R-peak detection, or may appear cleaner after denoising without guaranteeing reliable beat timing. Similarly, acceptable spectral properties do not compensate for absent contact. Therefore, ECG usability for HR estimation should not be determined by a single quality descriptor, but by combining evidence on contact availability, beat reliability, morphology, and SQI support.

These considerations highlight the need for a task-specific ECG usability framework rather than a binary quality classification. This is particularly relevant for cockpit-integrated sensing, where the sensing interface is not continuously attached to the body but depends on the pilot’s natural interaction with the instrumented controls. Thus, the present study addresses this need by proposing a window-based framework for assessing the quality and usability of simultaneous wECG and iECG acquired during simulated flight. Data were collected in an Airbus A320 simulator from 14 participants (4 experienced pilots and 10 novices), using a three-electrode wearable ECG configuration and custom conductive electrodes integrated into cockpit controls. The framework combines evidence on contact availability, R-peak reliability, PQRST morphology, and SQI support to assign each window to task-specific usability classes. The aim is to determine whether each ECG segment preserves sufficient physiological information for HR-oriented cockpit monitoring, avoiding the unnecessary rejection of HR-usable windows while excluding segments that are physiologically unreliable.

## 2. Materials and Methods

### 2.1. Experimental Setup and Protocol

**Figure 1:**
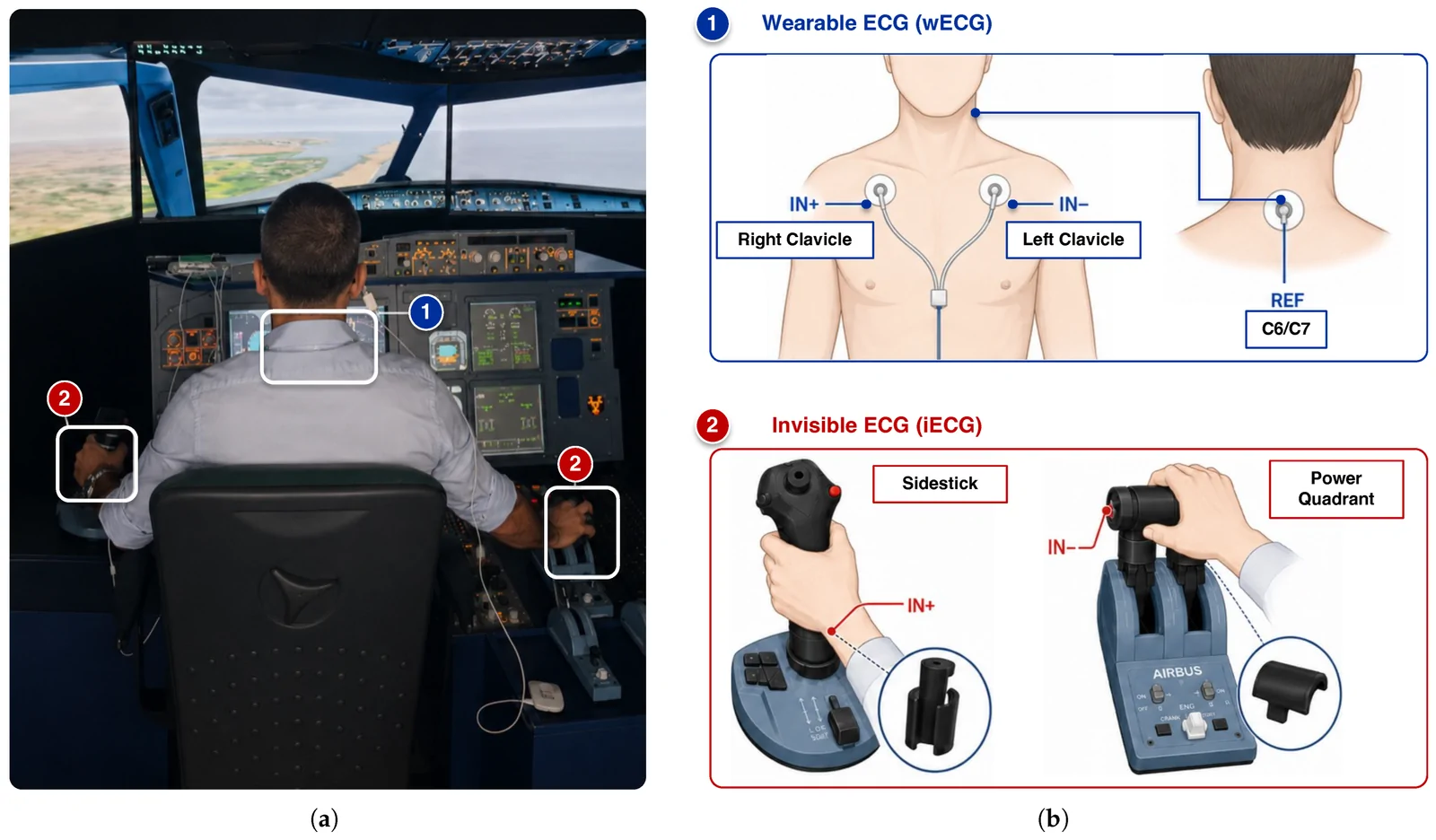
(**a**) Experimental setup with a volunteer in the Airbus A320 flight simulator. (**b**) Sensing modalities: (1) commercial pre-gelled electrodes used for wECG; (2) custom 3D-printed conductive electrodes externally mounted on the sidestick and power quadrant for iECG sensing.

ECG data were acquired in an Airbus A320 flight simulator at the Avionics Laboratory of Instituto Superior Técnico (IST, Lisbon, Portugal). The simulator is built on the X-Plane 11 platform (Laminar Research, South Burlington, VT, USA) with cockpit hardware from SKALARKI electronics Ltd. (Birmingham, UK), reproducing a realistic single-aisle cockpit environment suitable for controlled physiological data acquisition [8] (Figure 1a). Using the Multipurpose Control and Display Unit (MCDU; SKALARKI electronics Ltd., Birmingham, UK), a standardized route was pre-programmed identically for all participants, departing from Beja Airport (ICAO: LPBJ, runway 19L) and arriving at Faro Airport (ICAO: LPFR, runway 28), in southern Portugal. Take-off and landing were flown manually, as the most cognitively demanding segments, while the cruise was managed by the autopilot. The flight was conducted below 10,000 ft (3048 m) under clear weather and without turbulence or abnormal events, so that interaction with the controls remained consistent with standard “sterile cockpit” operation, in which the pilot keeps the hands on the controls during the critical phases. Although the predefined route corresponded to a nominal flight time of approximately 30 min, the effective recording retained for analysis varied across participants; after temporal alignment and trimming to the interval common to both ECG modalities from take-off to landing, the analyzed recording length was 19.7±5.7 min (mean ± SD; range 12.9–33.0 min).

Because the iECG relies on the surfaces that the pilot naturally and continuously touches while flying, the cockpit controls were first reviewed to identify suitable hosting sites. Among the manual flight controls, the sidestick and the throttle (power) quadrant are continuously gripped during manual operation and were therefore selected to embed the iECG electrodes. The analyzed cohort comprised 14 participants, including 4 experienced pilots and 10 novices, as summarized in Table 1. Data collection was approved by the Ethics Committee of IST (ref. 17/2025 CE-IST, 9 June 2025), and all participants provided written informed consent.

### 2.2. Sensing Modalities and System Architecture

Two ECG modalities were recorded simultaneously, to enable a paired comparison between a wearable reference and the invisible approach (Figure 1b), as described in our previous work [8].

The acquisition system was built around a BITalino (r)evolution Core BT board (PLUX Wireless Biosignals, Lisbon, Portugal), used as a compact multichannel biosignal acquisition platform. The board integrates analogue input channels, analogue-to-digital conversion, wireless Bluetooth transmission, and battery-powered operation, enabling physiological data recording inside the simulator without constraining the pilot’s movements. The BITalino device was connected to two ECG front-end sensors, one assigned to the wearable configuration and one assigned to the invisible configuration. In the complete experimental setup, the wearable ECG and invisible ECG were acquired on separate analogue channels, allowing synchronous recording of both modalities under the same flight conditions. Raw data were streamed in real time to OpenSignals (r)evolution v2.2.5 (PLUX Wireless Biosignals, Lisbon, Portugal) at 1000 Hz, which was used for signal visualization, acquisition control, and data export for offline processing.and data export for offline processing.

The ECG front-end measures the differential biopotential between the sensing electrodes and provides an analogue output compatible with the BITalino input range. For the wearable configuration, the ECG sensor was connected to a three-lead cable, including two measurement electrodes and one reference electrode. For the invisible configuration, the ECG sensor was connected to the two 3D-printed electrodes embedded in the cockpit controls, enabling ECG acquisition through the pilot’s natural hand contact with the sidestick and power quadrant. In both cases, the acquired analogue signal was digitized by the BITalino board and stored as raw analogue-to-digital converter counts.

The wECG was acquired in a three-electrode configuration using commercial pre-gelled Ag/AgCl electrodes (Kendall Arbo H124SG, Covidien Ltd., Gosport, UK), with two clavicular electrodes and one cervical reference. Specifically, IN+ was positioned on the right clavicle, IN– on the left clavicle, and the reference electrode at C6/C7, according to the placement adopted in the experimental protocol. These disposable, gel-based electrodes provide a stable, low-impedance skin interface and were used as the wearable body-contact reference for the paired comparison with iECG.

The iECG was recorded through custom electrodes 3D-printed in electrically conductive composite polylactic acid (PLA) filament (ProtoPasta, ProtoPlant Inc., Vancouver, WA, USA) and embedded in the cockpit controls, namely the power quadrant and the sidestick. Two geometries were modeled in SolidWorks 2023 (Dassault Systèmes SolidWorks Corp., Waltham, MA, USA) one tailored to the sidestick and one to the power quadrant, so that each electrode matched the ergonomics of the control surface and the pilot’s natural grip (Figure 1b). Conductive-filament electrodes provide a practical route for embedding dry sensing interfaces into customized objects [38,39]. However, compared with gel-based wearable electrodes, dry integrated electrodes are more affected by contact variability and motion artifacts, motivating the contact-aware usability assessment described in Section 2.3.

### 2.3. ECG Data Processing and Task-Specific Usability Assessment

Invisible ECG acquisition through 3D-printed conductive PLA electrodes embedded in cockpit controls introduces degradation patterns that differ qualitatively from those of conventional adhesive gel-based wearable electrodes. Intermittent hand contact with the instrumented controls, variable grip pressure, and the absence of conductive gel may produce full-contact, partial-contact, and no-contact conditions within the same recording session. Therefore, contact availability was assessed before downstream ECG quality, so that windows without contact were interpreted as lack of physiological information rather than as low-quality ECG.

**Figure 2:**
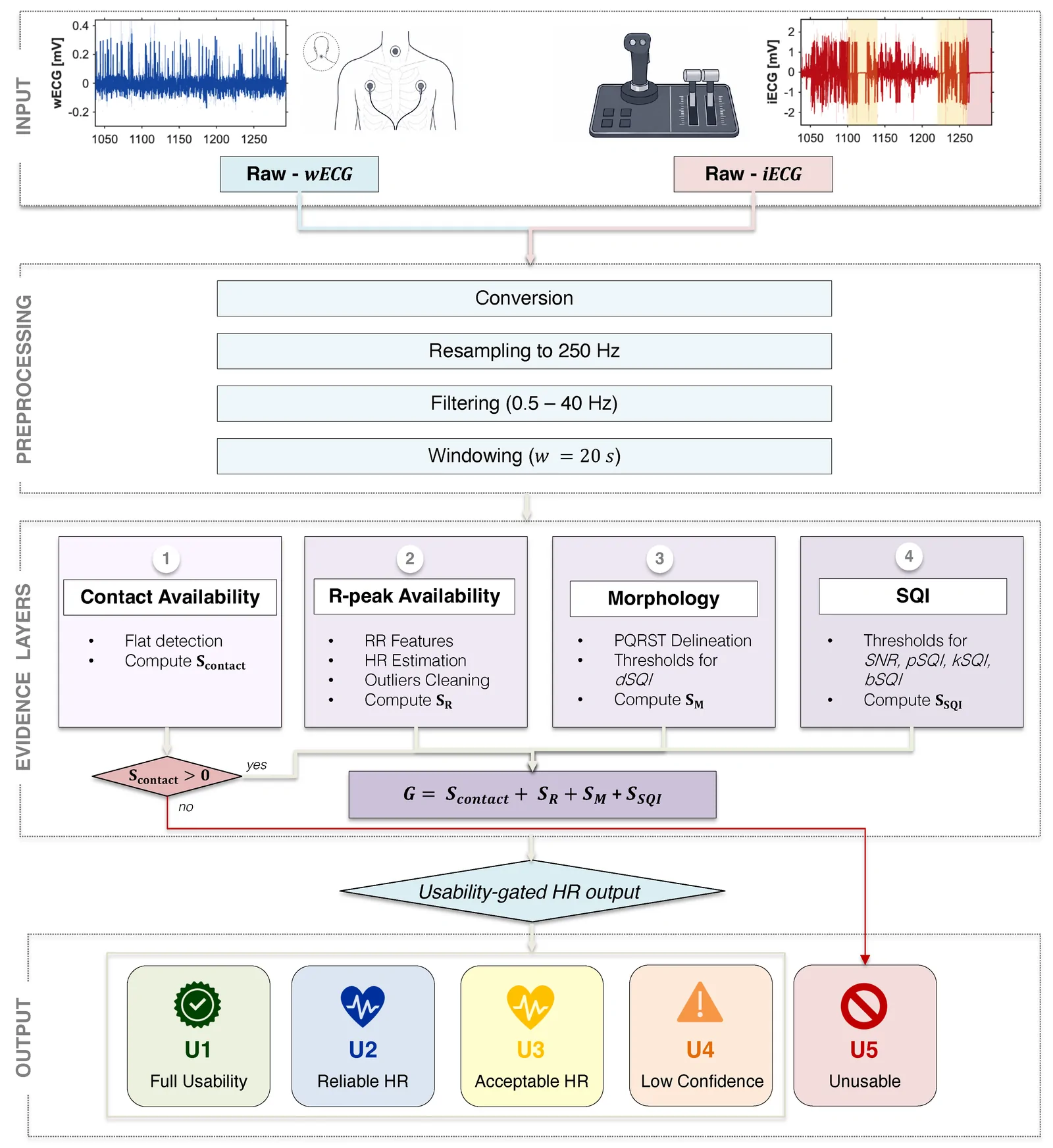
Overview of the task-specific ECG usability framework. Raw wECG and iECG signals are converted into physical units, preprocessed, and segmented into analysis windows. Four evidence layers are then extracted, namely, contact availability, R-peak reliability, morphology evidence, and SQI support. The resulting sub-scores are fused into a global usability score and mapped to five task-specific ECG usability classes.

The complete temporally aligned recording was processed; segmentation was used only to define local analysis units and did not select isolated ECG cycles or discard the remainder of the recording. Consecutive non-overlapping 20s windows were analyzed across the full aligned interval. This duration was selected as a compromise between retaining multiple cardiac cycles for local HR estimation, R-peak reliability assessment, morphology and SQI computation, while preserving the temporal resolution needed to capture changes in hand–electrode contact (consistently with [29,30,32]). The ECG processing was organized as a stepwise procedure moving from raw signal preparation to evidence extraction and final class assignment. First, Section 2.3.1 describes signal conversion, temporal alignment, filtering, and window segmentation. Section 2.3.2, Section 2.3.3, Section 2.3.4 and Section 2.3.5 then introduce the four evidence layers used to characterize each window: contact availability, R-peak reliability, morphology evidence, and SQI support. Finally, Section 2.3.6 describes how the four layer-specific scores are fused into a global score *G* and mapped to the final task-specific usability classes U1–U5. An overview of the complete task-specific ECG usability framework is provided in Figure 2.

#### 2.3.1. Signal Conversion and Preprocessing

Offline ECG processing and data analyses were performed in MATLAB R2025b (The MathWorks, Inc., Natick, MA, USA). Raw samples were converted from analogue-to-digital converter (ADC) counts to physical amplitude values in millivolts, because amplitude-dependent operations (i.e., flat segment detection and R-peak amplitude variability) must be performed in physical units rather than device-dependent counts. For a 10-bit acquisition channel with operating voltage $V_{cc}=3.3\;V$ and ECG sensor gain, $G_{ECG}=1100$:

(1) $$ECG\;\left[V\right]=\frac{\left(\frac{ADC}{2^{n}}-\frac{1}{2}\right)V_{cc}}{G_{ECG}},\;ECG\;\left[\mathrm{mV}\right]=ECG\;\left[V\right]\cdot 1000,$$

Here, ADC denotes the dimensionless digital value sampled from the acquisition channel and n=10 is the ADC resolution; $V_{cc}=3.3\;V$ and $G_{ECG}=1100$ are the operating voltage and ECG-sensor gain, respectively. Equation (1) follows the BITalino ECG transfer function. Converted signals were temporally aligned and resampled from 1000 to 250Hz [40]. Both ECG modalities were acquired through the same BITalino ECG front end, characterized by a nominal analogue bandwidth of 0.5–40Hz [41]. The aligned recordings were then partitioned into consecutive non-overlapping 20s windows spanning the complete common recording interval. Within each window, contact-related flat segments were handled as described in Section 2.3.2; analyzable windows were subsequently z-normalized by subtracting the window mean and dividing by the window standard deviation before R-peak, morphology, and SQI analysis.

#### 2.3.2. Evidence Layer 1—Contact Availability

The first evidence layer quantified electrode contact availability and contributed the sub-score $S_{contact}\in \left\{0,\;1,\;2\right\}$. This layer was evaluated prior to all downstream processing, because absent electrode contact represents signal unavailability rather than degraded signal quality: the two conditions have different physical origins and require different responses. Flat or near-flat segments were detected in millivolts using two complementary criteria: a sample-to-sample amplitude variation below 0.005mV, or a local amplitude range below 0.05mV within 500ms sub-windows. A segment was declared flat only when either condition persisted for at least 2s, to avoid classifying isolated low-amplitude fluctuations as contact loss. The total flat duration $T_{flat}$ within each window was mapped to

(2) $$S_{contact}=\left\{\begin{array}{cc} 2, & T_{flat}<2\;s\;(\mathrm{full}\;\mathrm{contact}),\\ 1, & 2\;s\leq T_{flat}<8\;s\;(\mathrm{partial}\;\mathrm{contact}),\\ 0, & T_{flat}\geq 8\;s\;(\mathrm{no}\;\mathrm{contact}). \end{array}\right.$$

Windows with $S_{contact}=0$ were unconditionally assigned to class U5 before computing *G*, because any R-peaks or spectral features extracted from a flat signal are electrical artifacts rather than cardiac activity. For the wearable ECG, adhesive gel-based electrodes maintain stable skin-contact throughout the protocol, so $S_{contact}=2$ for all wECG windows by construction: the contact layer is therefore informative exclusively for the iECG channel.

#### 2.3.3. Evidence Layer 2—R-Peak Reliability and Candidate HR Estimation

Even when PQRST morphology is incomplete, R-peak timing may remain sufficiently stable for HR computation. The second layer therefore assessed the temporal reliability and physiological plausibility of the detected R-peak sequence, providing the sub-score $S_{R}\in \left\{0,\;1,\;2,\;3\right\}$.

Within each analysis window, R-peak detection followed the QRS-enhancement procedure previously adopted in our work [1]. The z-normalized ECG was band-pass filtered using a first-order Butterworth filter between 20 and 30Hz; the Hilbert envelope was then computed and used for R-peak localization. This QRS-enhanced representation was used for R-peak timing, whereas morphology and SQI features were computed from the z-normalized broad-band ECG window. Three reliability statistics were computed within each window: the total number of detected R peaks $N_{R}$; the fraction of RR intervals within the physiologically plausible range 300–2000 ms, corresponding to 30–200 bpm, denoted as $P_{RR}$; and the coefficient of variation of R-peak amplitude, $CV_{amp}$. The latter was included to penalize detections destabilized by variable contact pressure or motion artifacts. Let $N_{RR}$ denote the number of RR intervals in the window and $N_{RR,\mathrm{valid}}$ the number satisfying $300\leq RR_{i}\leq 2000\;\mathrm{ms}$. Let $a_{R,j}=\left|x_{z}\left(r_{j}\right)\right|$ denote the absolute amplitude of the *j*-th detected R peak in the z-normalized ECG window $x_{z}$. The two statistics were computed as

(3) $$P_{RR}=\frac{N_{RR,\mathrm{valid}}}{N_{RR}},\;$$

(4) $$CV_{amp}=\frac{\sigma (a_{R})}{\mu (a_{R})},\;a_{R}=\left[a_{R,1},\ldots ,a_{R,N_{R}}\right],$$

where $\mu (a_{R})$ and $\sigma (a_{R})$ denote the mean and standard deviation of the absolute detected R-peak amplitudes within the window, respectively. In the implementation used to generate the reported results, the numerical R-peak sub-score was based on the number of detected R peaks and the fraction of physiologically plausible RR intervals. $CV_{amp}$ was retained as an auxiliary descriptor of R-peak amplitude stability, rather than as an additional term in $S_{R}$. The corrected score definition is

(5) $$S_{R}=\left\{\begin{array}{cc} 3, & N_{R}\geq 12\;\;\mathrm{and}\;\;P_{RR}\geq 0.85,\\ 2, & N_{R}\geq 8\;\;\mathrm{and}\;\;P_{RR}\geq 0.70,\\ 1, & N_{R}\geq 4,\\ 0, & \mathrm{otherwise}. \end{array}\right.\;$$

The $P_{RR}$ thresholds of 0.85 and 0.70 were fixed operating criteria for grading the proportion of physiologically plausible intervals within each 20 s window; they were not estimated from the present cohort and should not be interpreted as universal physiological cut-offs. The $CV_{amp}$ threshold of 1.20 was used only as a permissive auxiliary guard against gross R-peak amplitude instability in the internal reliability diagnostic and does not contribute directly to $S_{R}$ or to the global score *G*.

Before HR agreement analysis, RR intervals were cleaned using an iterative beat-to-beat consistency criterion [1]. Specifically, RR intervals showing an absolute consecutive variation greater than 50ms were removed over three iterations. For each window, RR intervals were first defined as

(6) $$RR_{i}=t_{R,i+1}-t_{R,i},$$

where $t_{R,i}$ is the occurrence time of the *i*-th detected R peak. The retained RR values form a vector, denoted by ${\mathrm{RR}}_{\mathrm{clean}}$. After beat-to-beat consistency filtering, it was defined as

(7) $${\mathrm{RR}}_{\mathrm{clean}}=\left\{RR_{i}:\left|RR_{i}-RR_{i-1}\right|\leq 50\;\mathrm{ms}\;\mathrm{after}\;\mathrm{iterative}\;\mathrm{filtering}\right\}.\;$$

A candidate window-level HR estimate was then computed as

(8) $$HR_{\mathrm{cand}}=\frac{60}{\mathrm{median}\left({\mathrm{RR}}_{\mathrm{clean}}\right)}.\;$$

This estimate was computed before score fusion because R-peak timing directly contributes to the $S_{R}$ reliability layer. However, $HR_{\mathrm{cand}}$ was retained as a usable physiological output only after the final classification.

#### 2.3.4. Evidence Layer 3—Morphology Evidence

Complete PQRST morphology is required for waveform-dependent analysis, whereas HR estimation depends primarily on reliable R-peak timing. The third layer assessed waveform morphology around the detected cardiac beats, contributing $S_{M}\in \left\{0,\;1,\;2,\;3\right\}$. Unlike $S_{R}$, which evaluates the amount and temporal plausibility of detected R-peak information, $S_{M}$ evaluates whether the waveform surrounding those detected beats preserves sufficient P–QRS–T structure for morphology-dependent analysis.

The dynamic signal quality index (dSQI) [33,34] was adopted as the primary morphology indicator. dSQI quantifies beat-level time–frequency morphology similarity by comparing each detected beat to an adaptive window template, producing a value in [0, 1] that reflects the consistency and completeness of the PQRST waveform. However, windows classified as dSQI-Medium or dSQI-Low may still exhibit a clearly identifiable R-peak even when the surrounding waveform is degraded. Discarding these windows on the basis of dSQI alone would eliminate segments that retain sufficient information for HR estimation. The graded $S_{M}$ score was designed precisely to capture this distinction: it awards the highest value to windows with both high dSQI and confirmed PQRST delineation, and preserves R-peak-only windows at the lower end.

PQRST delineation was performed around each detected R-peak, accepting a beat as morphologically complete when the temporal order P<Q<R<S<T was preserved and the main intervals fell within broad physiological bounds: PR 80–260 ms, QRS 45–150 ms, QT 220–600 ms [26,27]. The sub-score was

(9) $$S_{M}=\left\{\begin{array}{cc} 3, & dSQI\geq 0.80\;\;\mathrm{and}\;\;N_{PQRST}\geq 3,\\ 2, & dSQI\geq 0.60\;\;\mathrm{and}\;\;N_{PQRST}\geq 2,\\ 1, & \text{R-peaks}\;\mathrm{present},\;\mathrm{PQRST}\;\mathrm{incomplete},\\ 0, & \mathrm{otherwise}, \end{array}\right.$$

where $N_{PQRST}$ is the count of morphologically complete beats. dSQI is used here exclusively as a morphology grading criterion within $S_{M}$; it is excluded from the SQI support layer (Layer 4) to avoid weighting the same morphological property twice in the additive sum *G*.

#### 2.3.5. Evidence Layer 4—SQI Support

No single SQI captures all relevant ECG degradation modes simultaneously. Baseline wander, power-line interference, broadband noise, motion artifacts, and variable electrode pressure alter the spectral distribution, amplitude statistics, and beat-to-beat consistency of the signal in different and often co-occurring ways. For this reason, four complementary SQIs were selected as the fourth evidence layer, each describing a different aspect of signal quality not fully captured by the morphology-oriented dSQI.

Specifically, the SNR was used to quantify spectral cleanliness, by comparing the power of the ECG band with the power of the out-of-band noise. The power-spectrum SQI (pSQI) was used to quantify the relative concentration of signal power within the QRS frequency band, which is particularly relevant for R-peak detection and HR estimation. The kurtosis SQI (kSQI) was used to describe the impulsiveness or peakedness of the ECG window, providing indirect evidence of sharp R-peak components. Finally, the beat-consistency SQI (bSQI) was used to quantify beat-to-beat waveform repeatability, by computing the Pearson correlation coefficient between each individual beat and the average beat template, and then taking the median of the resulting beat-wise correlation coefficients.

Mathematical definitions and thresholds for all indices are provided in Table 2. The direction and expected ranges of the SQIs were informed by established ECG quality literature [29,30,31,32], whereas the exact cut-points were fixed as rule-based operating categories for this deterministic framework and were not learned by a classifier or estimated from the present cohort during score fusion. Accordingly, these thresholds should be interpreted as engineering usability bins rather than universal clinical boundaries. SNR values of 5 and 10 dB separate low, intermediate, and comparatively cleaner spectral conditions; the central pSQI interval 0.50–0.80 represents the intended QRS-band power concentration, with adjacent ranges treated as intermediate; for kSQI, 3 corresponds to the Gaussian-reference kurtosis and values at or above 5 indicate more pronounced peakedness; and bSQI thresholds of 0.60 and 0.80 provide graded levels of beat-template repeatability.Each SQI was then classified as High, Medium, or Low according to Table 2. The SQI sub-score was then assigned by majority vote: $S_{SQI}=2$ when three or four indices were classified as High or Medium, $S_{SQI}=1$ when exactly two indices were classified as High or Medium, and $S_{SQI}=0$ otherwise.

#### 2.3.6. Score Fusion and Five-Class Usability Output

The four sub-scores were summed into a global usability score:

(10) $$G=S_{contact}+S_{R}+S_{M}+S_{SQI}\;\in \left[0,10\right].$$

The additive structure is used as a deterministic evidence-fusion rule across complementary layers; the layers are not assumed to be statistically independent and may share information, particularly at the R-peak level. The formulation is fully deterministic, requires no labeled training data, and imposes negligible computational overhead.

Classification proceeds in two steps. First, the contact gate: windows with $S_{contact}=0$ are assigned unconditionally to U5 before *G* is computed, because any features derived from a flat signal are artifactual. For all windows with $S_{contact}\geq 1$, *G* is then mapped to one of four active classes:

- U1 — Full usability: G≥9, $S_{M}=3$, $S_{R}=3$. Full PQRST morphology confirmed; HR, HRV, and waveform analysis supported.
- U2 — Reliable HR: G≥7, $S_{R}\geq 2$. Reliable HR and RR estimation; full morphology not guaranteed.
- U3 — Acceptable HR: G≥5, $S_{R}\geq 2$. HR available with reduced confidence; HRV not supported.
- U4 — Low confidence: all remaining windows with $S_{contact}\geq 1$. Signal physically present but insufficient for reliable HR estimation.
- U5 — Unusable: all windows with $S_{contact}=0$.

U5 denotes signal absence (no electrode contact); U4 denotes signal presence with insufficient quality. This distinction is physically grounded and clinically meaningful: U5 windows cannot be improved by any signal-processing step, whereas U4 windows may in principle benefit from adaptive filtering or artifact rejection. The wearable ECG never enters U5, as adhesive electrodes maintain contact throughout. The five-class output extends prior binary and three-state frameworks [29,30] by enabling graded recovery: windows that dSQI alone would reject may be retained as U2 or U3 when R-peak timing remains reliable.

To empirically assess whether the additive score provides a meaningful ordering of HR usability, the iECG global score *G* was compared with the absolute HR error relative to the temporally paired wECG window. Monotonicity was assessed using Spearman rank correlation and by examining HR mean absolute error (MAE) across increasing *G*-score ranges.

## 3. Results

The results are organized according to the main components of the proposed framework. First, contact availability is analyzed to distinguish windows with full, partial, or absent electrode contact. Then, ECG morphology is assessed through dSQI and the individual SQI metrics. Window-level usability, channel concordance, and the recovery of windows rejected by single-metric criteria are subsequently evaluated. Finally, the analysis focuses on RR-interval estimation and HR agreement between wearable and invisible ECG.

### 3.1. Contact Availability and Signal Accessibility

The wECG provided continuous signal accessibility throughout the recordings, consistent with its adhesive gel-based electrode configuration. Accordingly, all wECG windows were classified as full-contact windows ($S_{\mathrm{contact}}=2$), and no further contact stratification was required for this channel. By contrast, the iECG channel relied on external hand contact with the instrumented cockpit controls, making signal accessibility inherently dependent on the interaction between the participant and the acquisition interface. For this reason, iECG contact availability analyzed on a window-by-window basis for each participant and according to expertise group are reported in Table 3.

Table 3 reports the contact distribution for the iECG channel only, showing also the last column as reporting accessible windows, defined as all windows with $S_{\mathrm{contact}}>0$.

Overall, the iECG channel showed high but participant-dependent signal accessibility. At the subject level (n=14), the mean ± SD within-subject proportions were 79.7±23.0% for full contact, 12.7±12.7% for partial contact, and 7.7±12.9% for no contact. At the pooled window level, 679/826 windows (82.2%) showed full contact, 96/826 (11.6%) partial contact, and 51/826 (6.2%) no contact; thus, 775/826 iECG windows (93.8%) were accessible ($S_{\mathrm{contact}}>0$).

A marked group-associated pattern was observed. At the subject level, pilots (n=4) showed 53.3±21.6% full-contact, 24.9±8.4% partial-contact, and 21.8±17.4% no-contact windows (mean ± SD across subjects), whereas novices (n=10) showed 90.2±13.3%, 7.8±10.8%, and 2.0±3.8%, respectively. At the pooled window level, full contact accounted for 109/193 pilot windows (56.5%) and 570/633 novice windows (90.0%); partial or missing contact accounted for 84/193 pilot windows (43.5%) and 63/633 novice windows (10.0%). An exploratory subject-level exact permutation check supported the full-contact and no-contact group differences (exact p=0.004 and p=0.003, respectively; Holm-adjusted p=0.006 for both), with large Cliff’s effect sizes (δ=−0.90 and δ=0.90, respectively). These results confirm that iECG availability is mainly governed by the external-contact interface and by the way participants interact with the instrumented controls, rather than by ECG signal quality alone.

**Figure 3:**
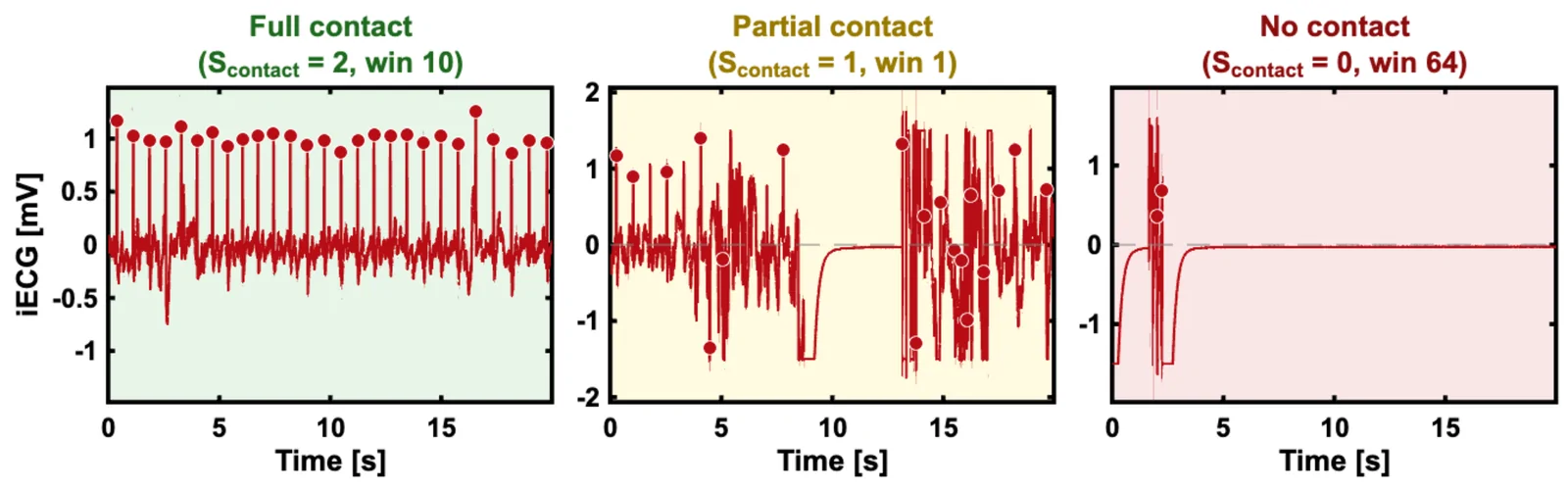
Representative iECG windows of S1 illustrating the contact states. The dashed line at zero marks the baseline reference.

Representative examples of the three iECG contact states are shown in Figure 3. Full-contact windows exhibit visible ECG activity, partial-contact windows alternate between informative segments and flat regions, and no-contact windows produce a quasi-flat trace in which apparent fluctuations should be interpreted as electrical artifacts rather than physiological information.

### 3.2. ECG Morphology Analysis

**Figure 4:**
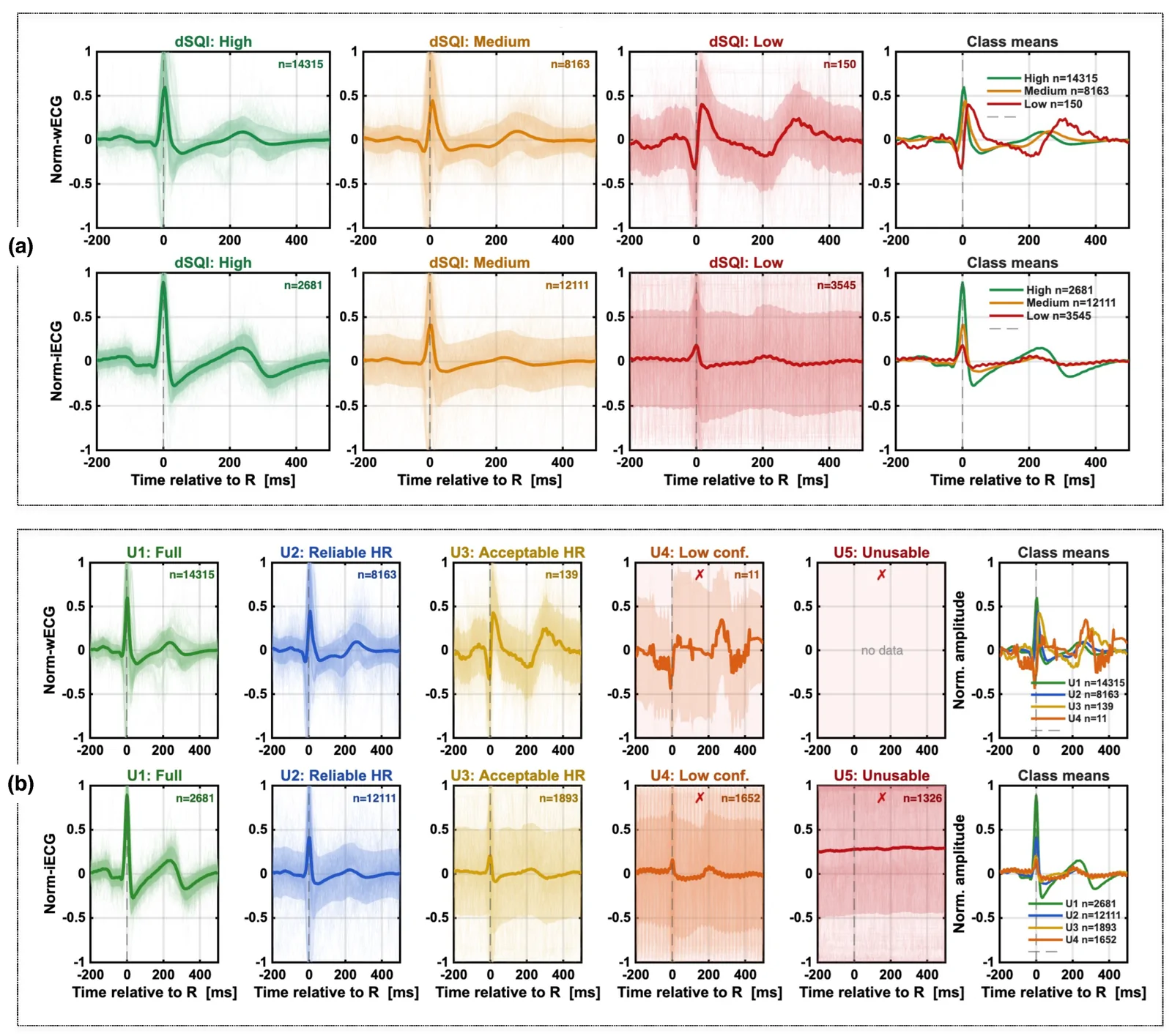
Beat-aligned ECG morphology for wECG and iECG. (**a**) Beats grouped according to dSQI category. (**b**) Beats grouped according to the final usability class assigned by the proposed framework. Solid lines represent the mean normalized waveform, shaded areas represent inter-beat variability, and *n* indicates the number of aligned beats in each group. The vertical gray dashed line marks the R-peak reference (0 ms).

ECG morphology was analyzed to verify whether morphology-based signal quality and task-specific usability provide equivalent or complementary information. To this aim, detected beats were aligned to the R-peak and grouped first according to the dSQI category and then according to the final usability class assigned by the proposed framework. The resulting beat-aligned morphologies are shown in Figure 4 for wECG and iECG.

The dSQI-based grouping confirmed that the wearable reference preserved substantially more stable morphology than the invisible channel (Figure 4a). In wECG, the Low-dSQI class included only 150 beats, compared with 14,315 and 8163 beats in the High and Medium classes, respectively. Thus, severe morphological degradation was rare in the adhesive skin-contact channel. In iECG, instead, the morphology distribution shifted markedly toward lower dSQI categories: only 2881 beats were classified as High, whereas 12,111 and 3545 beats were classified as Medium and Low, respectively. The number of Low-dSQI beats was therefore more than twenty times higher in iECG than in wECG, confirming the impact of the dry external-contact interface on waveform repeatability and PQRST stability.

Despite this degradation, low morphology quality did not necessarily correspond to loss of cardiac timing information. In the iECG Low-dSQI group, the average waveform still retained a visible R-peak component, although with larger beat-to-beat variability and less consistent P and T wave representation. This observation is relevant because the main target of the proposed framework is HR estimation, which depends primarily on reliable R-peak timing rather than on complete PQRST morphology. A purely morphology-based criterion would therefore be overly conservative for iECG, as it would reject segments that may still contain physiologically meaningful timing information.

The final usability classification confirmed this distinction (Figure 4b). In wECG, almost all beats were assigned to U1 or U2, with 14,315 beats in U1 and 8163 in U2, while only 139 and 11 beats were assigned to U3 and U4, respectively, and no beats were assigned to U5. This indicates that the wearable channel provided either full morphology or reliable HR usability for nearly the entire recording.

For iECG, the final classification showed a more nuanced behavior. While only 2881 beats were assigned to U1, 12,111 beats were classified as U2 and 8993 as U3. Therefore, a large fraction of iECG beats was retained as reliable or acceptable for HR estimation despite not always preserving full PQRST morphology. Conversely, 1652 beats were assigned to U4 and 1326 to U5, identifying segments in which low-confidence or missing physiological information justified rejection or cautious interpretation. Overall, the iECG classification was therefore not simply a degradation from U1 to unusable, but a redistribution of morphologically imperfect signals into task-specific usability levels.

### 3.3. Usability Classification and Concordance

**Figure 5:**
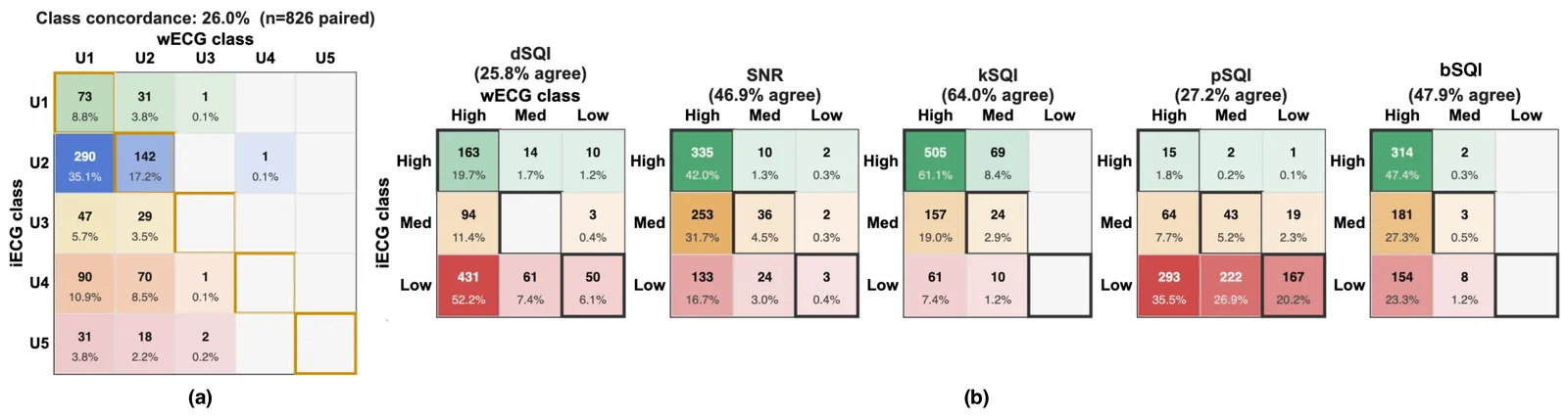
Window-level concordance between wECG and iECG. (**a**) Concordance matrix between final usability classes for temporally paired windows. (**b**) SQI-level concordance matrices between wECG and iECG for dSQI, SNR, kSQI, pSQI, and bSQI. Cell colors indicate the iECG usability class in panel (**a**) and the iECG SQI category in panel (**b**), while outlined diagonal cells denote concordant classifications between wECG and iECG.

The paired window-level concordance between wECG and iECG is reported in Figure 5a. The two channels showed limited class concordance, with matching usability classes in 26.0% of the 826 paired windows. This result was expected, as the wECG channel was acquired through stable adhesive electrodes, whereas the iECG channel depended on external hand contact with the cockpit controls. Accordingly, wECG windows were almost entirely assigned to high usability levels, with 531/826 windows classified as U1 and 290/826 as U2. In contrast, iECG showed a broader distribution across the five usability classes: 105 windows were assigned to U1, 433 to U2, 76 to U3, 161 to U4, and 51 to U5.

The dominant discordance pattern was therefore not random disagreement, but a systematic shift of iECG toward lower usability classes. The most frequent off-diagonal combination was wECG-U1/iECG-U2, accounting for 290 paired windows (35.1%). This indicates that, in many cases, the invisible channel did not preserve full morphology but still provided reliable HR usability. Conversely, iECG-U4 and iECG-U5 windows occurred mainly when the corresponding wECG window remained U1 or U2, confirming that these low-usability assignments were caused by the external-contact iECG interface rather than by a true absence of cardiac activity.

The SQI-level concordance analysis further supports this interpretation (Figure 5b). Agreement between wECG and iECG depended strongly on the metric considered, ranging from 25.8% for dSQI and 27.2% for pSQI to 64.0% for kSQI. This variability confirms that individual SQIs capture different degradation mechanisms and that no single metric is sufficient to determine task-specific usability in external-contact ECG. In particular, morphology- and spectrum-based SQIs frequently classified iECG windows as lower quality than the paired wECG windows, even when the final framework retained them as HR-usable.

### 3.4. Recovery from Single-Metric Rejection

To quantify the advantage of the proposed framework over conventional single-metric thresholding, the windows classified as Low by each individual SQI were identified and compared with the final usability classification. A window was considered recovered when it was assigned to U1, U2, or U3 by the complete framework, indicating that it still retained sufficient information for full ECG usability or HR-oriented monitoring. Results are reported in Table 4 and Table 5 for pilots and novices, respectively.

The recovery analysis shows that single-SQI rejection would discard a substantial number of windows that remain physiologically useful after multilayer assessment. This effect was particularly evident for iECG, where morphology and spectral indices were more frequently affected by external-contact variability. In novices, 416 iECG windows were classified as Low by dSQI alone, but 322 of them were recovered as U2 or U3 by the proposed framework. Similarly, pSQI classified 514 novice iECG windows as Low, of which 398 were recovered. These results indicate that poor single-metric quality does not necessarily imply loss of HR usability.

A different pattern was observed in pilots. Although iECG also showed high single-metric rejection rates, recovery percentages were lower than in novices. For example, among pilot iECG windows classified as Low by dSQI, 32/126 windows were recovered, while 46/168 windows classified as Low by pSQI were recovered. This suggests that, in pilots, low SQI values were more often associated with true contact-related degradation or unreliable timing, rather than with morphology degradation alone. Therefore, the framework does not indiscriminately recover low-quality windows; instead, it preserves only those supported by contact availability, R-peak reliability, morphology evidence, and complementary SQIs.

### 3.5. RR Consistency and Heart Rate Agreement

**Figure 6:**
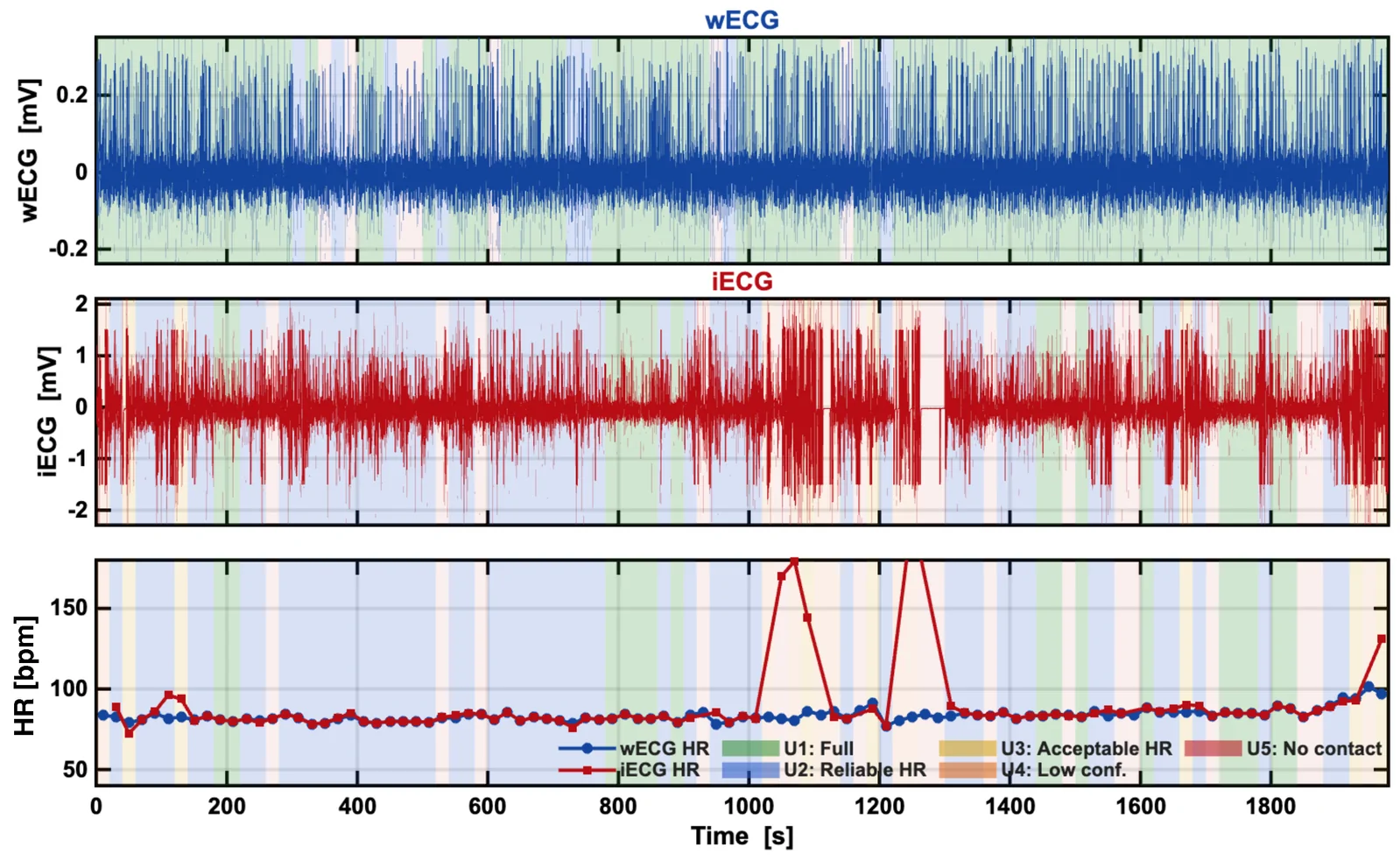
Single-participant example showing simultaneous wECG and iECG signals, HR estimates, and window-level usability classes.The legend reports the complete U1–U5 color coding of the usability framework for consistency.

Figure 6 shows a single-participant example used to illustrate how the proposed usability classes relate to the temporal behavior of the ECG signals and HR estimates. This example is not intended as a quantitative result, but provides visual context for the following RR consistency and HR agreement analyses. In particular, it shows how periods of degraded iECG signal morphology can coexist with HR estimates close to the wECG reference when R-peak timing remains stable, whereas larger deviations tend to appear during lower-usability intervals.

**Figure 7:**
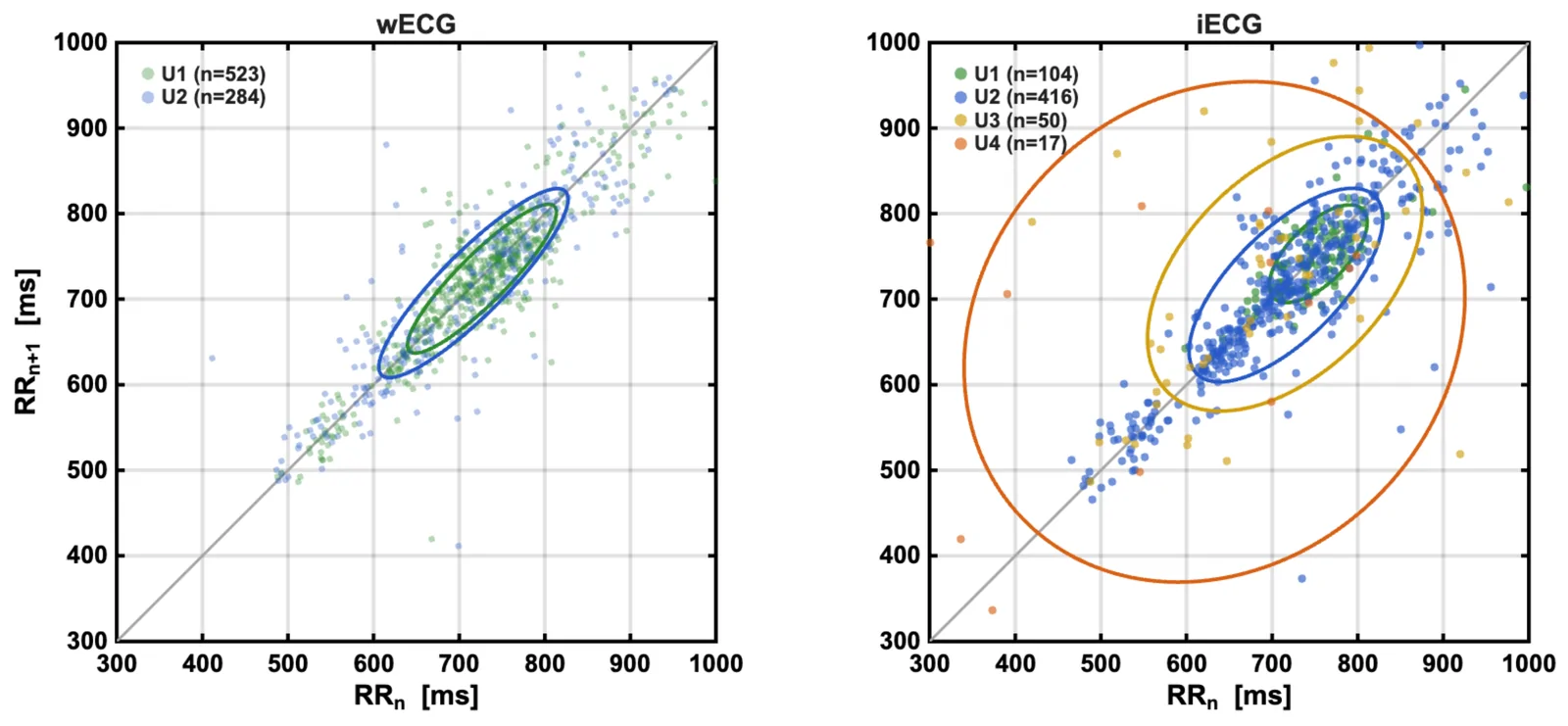
Poincaré plots of consecutive RR intervals for wECG and iECG, colored by usability class. Ellipses represent class-specific RR dispersion. U5 windows are excluded.

The physiological relevance of the proposed usability classes was evaluated by analyzing RR timing consistency and HR agreement between iECG and the paired wECG reference. As HR estimation depends primarily on the temporal stability of consecutive R-peaks, this analysis focused on windows for which a cleaned RR sequence could be obtained. Windows classified as U5 were excluded because they corresponded to no contact. U4 windows were retained only as low-confidence reference points, whereas the main HR agreement statistics were computed on windows classified as U1–U3. Poincaré plots of consecutive RR intervals are shown in Figure 7.

**Figure 8:**
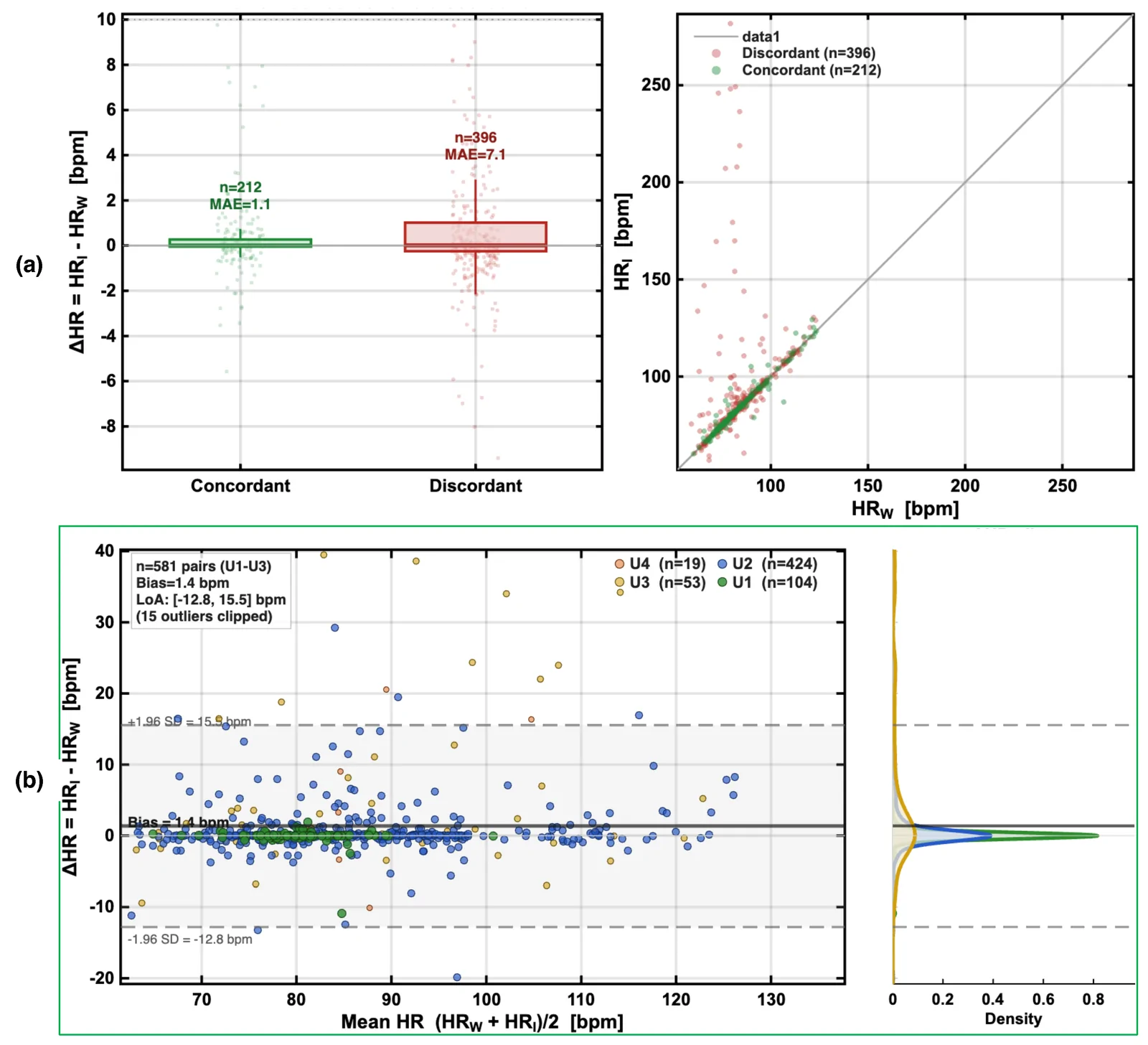
HR agreement between iECG and wECG according to the proposed usability framework. (**a**) **Left**: MAEs for concordant and discordant paired windows. **Right**: iECG-derived HR versus wECG-derived HR, with concordant and discordant windows highlighted separately. (**b**) Bland–Altman analysis for iECG windows classified as U1–U3.

In wECG, RR pairs were concentrated along the identity line and were mainly associated with U1 and U2, confirming the temporal stability of the wearable reference channel. In iECG, the RR distribution was broader and more class-dependent, reflecting the residual timing uncertainty introduced by the external-contact interface. However, U1 and U2 iECG points remained largely clustered around the identity line, indicating preserved beat-to-beat timing regularity in the classes intended for reliable HR estimation. In contrast, U3 showed increased dispersion, while U4 was associated with the widest variability. This confirms that the proposed usability classes provide a graded description of RR timing reliability, rather than a purely morphology-based signal quality label. The relationship between usability classification and HR estimation accuracy is reported in Figure 8a. First, the effect of class concordance between wECG and iECG was evaluated on paired HR estimates. Concordant windows, defined as temporally paired windows assigned to the same usability class in both channels, showed a mean absolute error (MAE) of 1.1 bpm (n=212). Conversely, discordant windows showed a substantially higher MAE of 7.1 bpm (n=396). This difference indicates that disagreement between wECG and iECG usability classes is not merely a classification mismatch, but is associated with reduced reliability of the iECG-derived HR estimate. The HR scatter plot further supports this result: concordant windows were tightly distributed around the identity line, whereas discordant windows included the largest deviations and clear overestimation episodes.

The overall agreement between iECG and wECG was then quantified using Bland–Altman analysis Figure 8b. Considering iECG windows classified as U1–U3, 581 paired HR estimates were retained. The mean bias was 1.4 bpm, indicating a small average overestimation of HR by iECG relative to wECG. The 95% limits of agreement ranged from −12.8 to 15.5 bpm. Most errors were concentrated around zero, particularly for U1 and U2 windows, while larger deviations were mainly associated with U3 and low-confidence U4 points. Therefore, the final classification provides a meaningful reliability gradient: U1 and U2 support reliable HR estimation, U3 retains acceptable but less stable HR information, U4 should be interpreted with low-confidence, and U5 corresponds to no contact.

The global score also showed a clear monotonic relationship with HR accuracy. Across 608 temporally paired windows with valid HR estimates, *G* was inversely associated with absolute HR error (Spearman’s ρ=−0.561, p<0.001). Pooled HR MAE decreased from 65.3 bpm for G=3–4, to 15.2 bpm for G=5–6, 1.62 bpm for G=7–8, and 0.50 bpm for G=9–10, with no upward transition across consecutive score ranges.

Overall, these results confirm the task-specific nature of the proposed framework. The classification does not only describe ECG waveform quality, but also indicates how confidently iECG can be used for HR-oriented cockpit monitoring. In particular, windows with incomplete morphology can still provide accurate HR estimates when contact availability, R-peak reliability, and SQI support remain sufficient, whereas windows affected by class discordance, unstable RR timing, or no contact are correctly assigned to lower usability levels.

## 4. Discussion and Conclusions

This study proposed and evaluated a window-based ECG quality and usability framework for unobtrusive cockpit monitoring, using simultaneous wECG and cockpit-integrated iECG acquired during simulated flight. ECG quality and ECG usability should not be treated as equivalent concepts when the target application is HR-oriented monitoring. In particular, the results show that iECG windows may present incomplete or degraded PQRST morphology while still preserving sufficient R-peak timing information to support reliable or acceptable HR estimation. This distinction is especially relevant for external-contact interfaces, in which signal degradation is not only caused by classical ECG artifacts, but also by intermittent hand contact, variable grip pressure, and changes in the interaction between the user and the instrumented surface.

The first result concerns contact availability. Unlike wECG, which maintained stable contact through adhesive gel-based electrodes, iECG showed a clear dependence on the way participants interacted with the cockpit controls. Overall, 775/826 iECG windows were accessible ($S_{\mathrm{contact}}>0$), corresponding to 93.8% of the analyzed windows. However, this global availability masked a marked group-associated pattern. Pilots showed a higher proportion of partial or missing contact than novices, with 43.5% of pilot windows affected by partial or absent contact compared with 10.0% in novices. This result confirms that, for invisible ECG, signal availability is an interface-level condition that must be assessed before conventional signal quality. A no-contact segment does not represent a noisy ECG signal, but rather the absence of physiological coupling between the body and the sensing interface. Treating these windows as simply low-quality ECG would be misleading, because no signal-processing step can recover cardiac information when contact is absent. Conversely, partial-contact windows may still contain informative segments and should not be rejected a priori.

The exploratory subject-level statistical check supports a group-associated difference in contact quality within this sample, but it should not be interpreted as evidence of a causal effect of flight experience. The two groups also differed in age (18–30 years for novices and 40–60 years for pilots), and the present sample does not permit the independent contribution of experience and age to be separated. Participant-specific skin properties and skin–electrode impedance, as well as grip force, hand posture, perspiration, and contact dynamics, can influence the coupling of dry or externally integrated electrodes [15,39]. Accordingly, larger age-balanced cohorts and more direct measurements of hand–electrode interaction will be required to determine whether experience itself contributes to the observed contact pattern.

This finding extends previous work on wearable, dry, textile, and capacitive ECG systems, where the acquisition interface is known to affect signal quality through electrode impedance, motion artifacts, contact pressure, and mechanical coupling [14,15,16,21,22,23]. In conventional wearable ECG, the electrode remains attached to the body, and most quality assessment methods focus on the degree of waveform corruption. In cockpit-integrated iECG, however, the electrode is embedded in an object naturally touched by the user. Therefore, contact is not guaranteed and can change within the same recording. The proposed contact layer directly addresses this additional source of uncertainty by separating signal accessibility from downstream ECG quality.

The morphology analysis further supports the need for a task-specific interpretation of ECG quality. The wECG channel preserved substantially more stable morphology, with only 150 beats assigned to the Low-dSQI group, whereas iECG showed a much larger number of Low-dSQI beats. This behavior is consistent with the expected limitations of dry and external-contact interfaces, in which P and T waves are more easily attenuated or distorted than the dominant QRS complex. However, the iECG Low-dSQI morphology still often retained a visible R-peak component. This observation is central to the proposed framework. Morphology-based indices such as dSQI are highly valuable when the objective is to identify stable PQRST morphology or to support diagnostic ECG interpretation [29,30,31,33,34]. Nevertheless, for cockpit monitoring, the primary output is often HR or RR-derived information rather than full waveform interpretation. In this context, rejecting a window only because PQRST morphology is incomplete may be overly conservative.

The final usability classification captured this distinction. In wECG, nearly all windows were assigned to U1 or U2, confirming that the wearable reference provided either full morphology or reliable HR usability across almost the entire recording. In iECG, instead, the windows were distributed across all usability classes. Although only 105 windows were classified as U1, 433 were assigned to U2 and 76 to U3. Therefore, many iECG windows that did not preserve full morphology still retained HR-oriented physiological utility. This result demonstrates that the proposed framework does not simply classify iECG as a degraded version of wECG. Rather, it identifies different levels of usable information: full morphology when available, reliable HR when R-peak timing remains stable, acceptable HR when confidence is reduced, low-confidence signal when interpretation is uncertain, and no-contact windows when physiological information is absent.

The low window-level concordance between wECG and iECG should therefore be interpreted in this context. Matching usability classes were observed in 26.0% of paired windows, but the dominant discordance pattern was wECG-U1/iECG-U2, accounting for 35.1% of paired windows. This indicates that the invisible channel frequently lost full morphological quality relative to the wearable reference, while still preserving reliable HR information. The SQI-level concordance analysis showed that individual quality indices were not interchangeable, with agreement between wECG and iECG ranging from 25.8% for dSQI to 64.0% for kSQI. This supports the view that no single SQI can fully describe the usability of external-contact ECG. Different indices capture different degradation mechanisms, and their interpretation depends on the physiological output.

The recovery analysis provides direct evidence of the added value of the multilayer framework. In novices, 416 iECG windows were classified as Low by dSQI alone, but 322 of them were recovered as U2 or U3 by the proposed classification. Similarly, 398/514 windows classified as Low by pSQI were recovered. These windows would have been discarded by a single-metric quality rule, despite retaining sufficient evidence for HR-oriented use. At the same time, the lower recovery observed in pilots shows that the framework is not indiscriminately permissive. Among pilot iECG windows classified as Low by dSQI, only 32/126 were recovered, and only 46/168 Low-pSQI windows were recovered. This suggests that, when low SQI values were associated with true contact loss or unstable R-peak timing, the framework correctly assigned windows to low-confidence or unusable classes. Therefore, the proposed approach preserves useful HR information without accepting windows that lack physiological reliability.

The HR agreement results confirm the physiological meaning of the usability classes. Concordant wECG–iECG windows showed an MAE of 1.1 bpm, whereas discordant windows showed a higher error of 7.1 bpm. This indicates that class disagreement was not merely a formal classification difference, but was associated with reduced reliability of the iECG-derived HR estimate. Bland–Altman analysis for iECG windows classified as U1–U3 showed a small mean bias of 1.4 bpm relative to wECG, with most errors concentrated around zero and larger deviations mainly associated with lower usability levels. These findings support the interpretation of U1 and U2 as reliable classes for HR estimation, U3 as an acceptable but less stable class, U4 as low-confidence, and U5 as true signal absence. The monotonic decrease in HR error with increasing *G* further supports the use of the additive score as an ordinal measure of task-specific HR usability, despite the expected conceptual overlap between some evidence layers.

From an application perspective, this graded interpretation is important for pilot-state monitoring. Previous studies have shown the relevance of HR and HRV-derived parameters for workload, stress, fatigue, vigilance, and autonomic regulation in aviation and other high-demand scenarios [9,10,11]. However, future cockpit monitoring systems must operate unobtrusively, and under realistic interaction conditions, a binary acceptable/unacceptable ECG decision may either discard too much data or retain unreliable windows.

Several limitations should be acknowledged. First, the study was conducted in a simulator and under a controlled flight scenario without turbulence, abnormal events, or strong environmental disturbances. Real-flight conditions may introduce additional motion artifacts, operational constraints, and behavioral variability. Second, the analyzed cohort was limited in size and included a small number of experienced pilots; therefore, the observed group differences in contact availability should be confirmed in larger and more balanced populations. Third, the wearable reference used a bilateral clavicular placement with a cervical reference at C6/C7 rather than a conventional precordial ECG configuration. Although the adhesive Ag/AgCl electrodes ensured stable skin contact, myoelectric contamination from nearby pectoral, shoulder, trapezius, or neck muscles cannot be entirely excluded, particularly during control manipulation. Validation against a conventional precordial reference configuration (e.g., V1–V2) would therefore provide a stricter reference for future morphology-dependent assessments. Future work should evaluate thresholds and windows robustness across different cockpit layouts, electrode geometries, flight phases, and user populations. Finally, the present analysis focused on HR-oriented monitoring. The use of iECG for HRV analysis or morphology-dependent ECG interpretation requires stricter validation and should be limited to windows with higher usability and stable RR dynamics.

In conclusion, the proposed framework shows that invisible ECG acquired from cockpit controls can provide physiologically meaningful HR information when signal availability, R-peak reliability, morphology evidence, and SQI support are jointly considered. By moving from a single-metric quality decision to a graded usability assessment, the framework represents a step toward unobtrusive, interpretable, and application-aware physiological monitoring in aviation.

## Figures and Tables

**Table 1:** Demographic and experience composition of the analyzed cohort.

Characteristic	Novices	Pilots	All
Participants (n)	10	4	14
Sex (male/female)	7/3	4/0	11/3
Age range (years)	18–30	40–60	18–60

**Table 2:** Signal quality indices used as complementary evidence in the task-specific ECG usability framework.

Metric	Formula	Description	Rationale	Thresholds
SNR	$SNR=10\log_{10}\;\left(\frac{P_{\mathrm{ECG}}}{P_{\mathrm{noise}}}\right)$	Spectral cleanliness.	Quantifies ECG band power relative to out-of-band noise [29,31,32].	High: ≥10 dB; Medium: 5–10 dB; Low: <5 dB.
pSQI	$pSQI=P_{\mathrm{QRS}}/P_{\mathrm{ECG}}$	QRS band power ratio.	Quantifies relative power in the QRS band for R-peak timing and HR estimation [29,31,32].	High: 0.50–0.80; Medium: 0.40–0.50 or 0.80–0.90; Low: otherwise.
kSQI	$kSQI=\frac{E[{\left(x_{z}-\mu_{z}\right)}^{4}]}{\sigma_{z}^{4}}$	R-peak impulsiveness.	Describes peakedness of the ECG window; supports detection of sharp R-peak components [29,31,32].	High: ≥5; Medium: 3–5; Low: <3.
bSQI	$bSQI={\mathrm{median}}_{i}\;\left(\rho (b_{i},\bar{b})\right)$	Median beat-template Pearson correlation.	Quantifies beat-to-beat waveform repeatability; identifies stable ECG morphology [29,30,31].	High: ≥0.80; Medium: 0.60–0.80; Low: <0.60.

*Note.* $P_{\mathrm{ECG}}$ denotes the signal power within the ECG analysis band, $P_{\mathrm{noise}}$ denotes the residual out-of-band noise power, and $P_{\mathrm{QRS}}$ denotes the power within the QRS frequency band. In the kSQI definition, $x_{z}$ is the normalized ECG window, while $\mu_{z}$ and $\sigma_{z}$ are its mean and standard deviation, respectively. In the bSQI definition, $b_{i}$ denotes the *i*-th beat waveform, $\bar{b}$ is the average beat template within the window, and ρ(·, ·) is the Pearson correlation coefficient. Thus, bSQI is the median summary of the set of beat-wise Pearson correlation coefficients; no median filtering is applied to the correlation itself.

**Table 3:** Per-subject iECG contact availability ($N_{\mathrm{tot}}$: total number of windows). Columns report windows included in $S_{\mathrm{contact}}=2$ (full contact), $S_{\mathrm{contact}}=1$ (partial contact), and $S_{\mathrm{contact}}=0$ (no contact). Individual subject rows report absolute window counts and within-subject percentages. Group and overall summary rows report pooled window-level counts together with the mean ± SD of the corresponding within-subject percentages (pilots: n=4; novices: n=10; overall: n=14).

Subject	Group	$N_{\mathrm{tot}}$	$S_{\mathrm{contact}}=2$	$S_{\mathrm{contact}}=1$	$S_{\mathrm{contact}}=0$	$S_{\mathrm{contact}}>0$
S1	Pilot	62	49 (79.0%)	8 (12.9%)	5 (8.1%)	57 (91.9%)
S2	Pilot	48	29 (60.4%)	14 (29.2%)	5 (10.4%)	43 (89.6%)
S3	Pilot	39	11 (28.2%)	10 (25.6%)	18 (46.2%)	21 (53.8%)
S4	Pilot	44	20 (45.5%)	14 (31.8%)	10 (22.7%)	34 (77.3%)
**Pilots total/mean ± SD**		**193**	**109 (53.3 ± 21.6%)**	**46 (24.9 ± 8.4%)**	**38 (21.8 ± 17.4%)**	**155 (78.2 ± 17.4%)**
S5	Novice	56	34 (60.7%)	15 (26.8%)	7 (12.5%)	49 (87.5%)
S6	Novice	57	56 (98.2%)	1 (1.8%)	0 (0.0%)	57 (100.0%)
S7	Novice	47	47 (100.0%)	0 (0.0%)	0 (0.0%)	47 (100.0%)
S8	Novice	49	48 (98.0%)	1 (2.0%)	0 (0.0%)	49 (100.0%)
S9	Novice	77	74 (96.1%)	1 (1.3%)	2 (2.6%)	75 (97.4%)
S10	Novice	99	90 (90.9%)	7 (7.1%)	2 (2.0%)	97 (98.0%)
S11	Novice	60	43 (71.7%)	17 (28.3%)	0 (0.0%)	60 (100.0%)
S12	Novice	55	55 (100.0%)	0 (0.0%)	0 (0.0%)	55 (100.0%)
S13	Novice	48	46 (95.8%)	1 (2.1%)	1 (2.1%)	47 (97.9%)
S14	Novice	85	77 (90.6%)	7 (8.2%)	1 (1.2%)	84 (98.8%)
**Novices total/mean ± SD**		**633**	**570 (90.2 ± 13.3%)**	**50 (7.8 ± 10.8%)**	**13 (2.0 ± 3.8%)**	**620 (98.0 ± 3.8%)**
**Overall/mean ± SD**		**826**	**679 (79.7 ± 23.0%)**	**96 (12.7 ± 12.7%)**	**51 (7.7 ± 12.9%)**	**775 (92.3 ± 12.9%)**

**Table 4:** Aggregated SQI-level recovery analysis for pilots (n=4). Absolute $N_{\mathrm{low}}$ and $N_{\mathrm{rec}}$ values are pooled window-level counts. Percentages reported as mean ± SD are calculated across subjects: $N_{\mathrm{low}}$ percentages are relative to each subject’s total windows, whereas $N_{\mathrm{rec}}$ percentages are relative to each subject’s low-quality windows for the corresponding SQI. U1/U2/U3 columns report mean percentages across subjects.

Channel	SQI	N_low_ (% ± SD)	N_rec_ (% ± SD)	U1 (%)	U2 (%)	U3 (%)
wECG	dSQI	7 (3.9 ± 6.5%)	7 (50.0 ± 57.7%)	0.0	41.7	8.3
	SNR	1 (0.6 ± 1.1%)	1 (25.0 ± 50.0%)	0.0	25.0	0.0
	kSQI	0 (0.0 ± 0.0%)	0 (0.0 ± 0.0%)	0.0	0.0	0.0
	pSQI	53 (29.4 ± 43.8%)	43 (67.2 ± 45.7%)	18.8	47.2	1.2
	bSQI	2 (1.1 ± 2.3%)	2 (25.0 ± 50.0%)	0.0	0.0	25.0
iECG	dSQI	126 (68.5 ± 24.1%)	32 (25.1 ± 6.7%)	0.0	20.0	5.1
	SNR	80 (43.3 ± 15.5%)	7 (7.5 ± 7.5%)	0.0	2.7	4.8
	kSQI	10 (5.9 ± 6.4%)	2 (16.7 ± 23.6%)	0.0	0.0	16.7
	pSQI	168 (88.4 ± 10.8%)	46 (26.6 ± 8.7%)	1.1	21.0	4.6
	bSQI	42 (22.3 ± 4.5%)	4 (9.6 ± 7.5%)	0.0	9.6	0.0

**Table 5:** Aggregated SQI-level recovery analysis for novices (n=10). Absolute $N_{\mathrm{low}}$ and $N_{\mathrm{rec}}$ values are pooled window-level counts. Percentages reported as mean ± SD are calculated across subjects: $N_{\mathrm{low}}$ percentages are relative to each subject’s total windows, whereas $N_{\mathrm{rec}}$ percentages are relative to each subject’s low-quality windows for the corresponding SQI. U1/U2/U3 columns report mean percentages across subjects.

Channel	SQI	N_low_ (% ± SD)	N_rec_ (% ± SD)	U1 (%)	U2 (%)	U3 (%)
wECG	dSQI	56 (10.4 ± 12.2%)	55 (49.1 ± 51.8%)	0.0	47.6	1.5
	SNR	7 (1.2 ± 2.4%)	6 (20.0 ± 42.2%)	0.0	17.5	2.5
	kSQI	0 (0.0 ± 0.0%)	0 (0.0 ± 0.0%)	0.0	0.0	0.0
	pSQI	134 (25.2 ± 29.8%)	108 (60.8 ± 34.1%)	23.9	35.9	1.0
	bSQI	0 (0.0 ± 0.0%)	0 (0.0 ± 0.0%)	0.0	0.0	0.0
iECG	dSQI	416 (66.2 ± 30.3%)	322 (81.6 ± 21.5%)	0.0	67.2	14.5
	SNR	80 (12.3 ± 14.7%)	38 (46.5 ± 42.4%)	0.0	12.6	34.0
	kSQI	61 (10.1 ± 13.3%)	28 (48.4 ± 30.5%)	0.0	0.0	48.4
	pSQI	514 (80.2 ± 26.5%)	398 (78.7 ± 19.3%)	4.2	62.1	12.4
	bSQI	121 (19.3 ± 12.4%)	81 (75.0 ± 30.9%)	0.0	60.8	14.2

## Data Availability

The data presented in this study are available on reasonable request from the corresponding author. The data are not publicly available due to privacy and ethical restrictions related to human-participant recordings.

## Abbreviations

The following abbreviations are used in this manuscript:

ADC	Analog-to-digital converter
APPLA	Associação dos Pilotos Portugueses de Linha Aérea
bSQI	Beat-consistency SQI
DEEC	Department of Electrical and Computer Engineering
dSQI	Dynamic signal quality index
ECG	Electrocardiography
FCT	Fundação para a Ciência e Tecnologia
HR	Heart rate
HRV	Heart rate variability
IATA	International Air Transport Association
IBI	Inter-beat interval
ICAO	International Civil Aviation Organization
iECG	Invisible ECG
kSQI	Kurtosis SQI
MAE	Mean absolute error
MCDU	Multipurpose Control and Display Unit
PLA	Polylactic Acid
pSQI	Power-spectrum SQI
RR	R-to-R interval
SD	Standard deviation
SNR	Signal-to-noise ratio
SQI	Signal quality index
wECG	Wearable ECG
